# Reduction in expression of the benign AR transcriptome is a hallmark of localised prostate cancer progression

**DOI:** 10.18632/oncotarget.8915

**Published:** 2016-04-22

**Authors:** Ryan Stuchbery, Geoff Macintyre, Marek Cmero, Laurence M. Harewood, Justin S. Peters, Anthony J. Costello, Christopher M. Hovens, Niall M. Corcoran

**Affiliations:** ^1^ Australian Prostate Cancer Research Centre Epworth, Richmond, VIC, Australia; ^2^ Department of Urology, Royal Melbourne Hospital and University of Melbourne, Parkville, VIC, Australia; ^3^ NICTA Victoria Research Laboratory, University of Melbourne, Parkville, VIC, Australia; ^4^ Department of Surgery, Royal Melbourne Hospital and University of Melbourne, Parkville, VIC, Australia

**Keywords:** prostate cancer, PSA screening, androgen signalling, prognostics

## Abstract

**Background:**

Despite the importance of androgen receptor (AR) signalling to prostate cancer development, little is known about how this signalling pathway changes with increasing grade and stage of the disease.

**Objective:**

To explore changes in the normal AR transcriptome in localised prostate cancer, and its relation to adverse pathological features and disease recurrence.

**Design:**

Publically accessible human prostate cancer expression arrays as well as RNA sequencing data from the prostate TCGA. Tumour associated PSA and PSAD were calculated for a large cohort of men (n=1108) undergoing prostatectomy.

**Outcome Measurements and Statistical Analysis:**

We performed a meta-analysis of the expression of an androgen-regulated gene set across datasets using Oncomine. Differential expression of selected genes in the prostate TCGA database was probed using the edgeR Bioconductor package. Changes in tumour PSA density with stage and grade were assessed by Student's t-test, and its association with biochemical recurrence explored by Kaplan-Meier curves and Cox regression.

**Results:**

Meta-analysis revealed a systematic decline in the expression of a previously identified benign prostate androgen-regulated gene set with increasing tumour grade, reaching significance in nine of 25 genes tested despite increasing AR expression. These results were confirmed in a large independent dataset from the TCGA. At the protein level, when serum PSA was corrected for tumour volume, significantly lower levels were observed with increasing tumour grade and stage, and predicted disease recurrence.

**Conclusions:**

Lower PSA secretion-per-tumour-volume is associated with increasing grade and stage of prostate cancer, has prognostic relevance, and reflects a systematic perturbation of androgen signalling.

## INTRODUCTION

The androgen receptor (AR) is a ligand-activated transcription factor that is central to the initiation and progression of prostate cancer. The importance of AR signalling is underscored by the clinical utility of castration in producing symptomatic relief and objective responses in men with advanced prostate cancer. During prostate development, AR activation is known to increase the expression of growth factors, transcription factors and signalling peptides that are involved in cell proliferation, resistance to apoptosis, invasion and migration, processes which are necessary for successful organogenesis and are subsequently exploited by cancer [1]. The expression of AR has been demonstrated to increase with increasing tumour grade and stage, as does the serum levels of prostate specific antigen (PSA), the product of the proto-typical androgen responsive gene KLK3 [2, 3]. In fact the positive relationship between increasing serum PSA and increasing tumour stage and grade informs all aspects of its usage in the clinical management of prostate cancer patients [4]. However, it has been recognised for some time that there exists a small cohort of patients with primary prostate cancer that remains localised to the prostate, in whom serum PSA does not accurately reflect the extent of their disease burden [5–8]. These ‘low-secreting’ tumours are reported to have a worse prognosis compared to grade and stage matched controls. This is supported by a number of studies indicating that the incidence of prostate cancer, including high-grade tumours, is significant in patients with serum PSA levels within the quoted normal range [9, 10].

Tomlins *et. al.* have previously reported that a cassette of genes normally up-regulated by AR signalling is in fact down-regulated during the progression from low-grade to high-grade disease [11]. However the cohort size was small (n=30), and the gene set used in the analysis was generated from the LNCaP cell line (derived from a treatment-refractory metastasis) stimulated with the synthetic androgen R1881, so the relevance to clinically localised prostate cancer under physiological conditions is unclear [11]. This univariable analysis was useful for generating the hypothesis that AR signalling decreases with increasing cancer grade but is not definitive. In a previous study we have observed that a decrease in PSA levels is correlated with increasing grade [12]. We were therefore interested in extending these findings to examine the expression of a patient-derived, normal AR transcriptome in different disease grades and stages and to examine what effect, if any, alterations in gene expression would have on protein expression of PSA and how this may affect clinical practice.

## RESULTS

**Figure 1:**
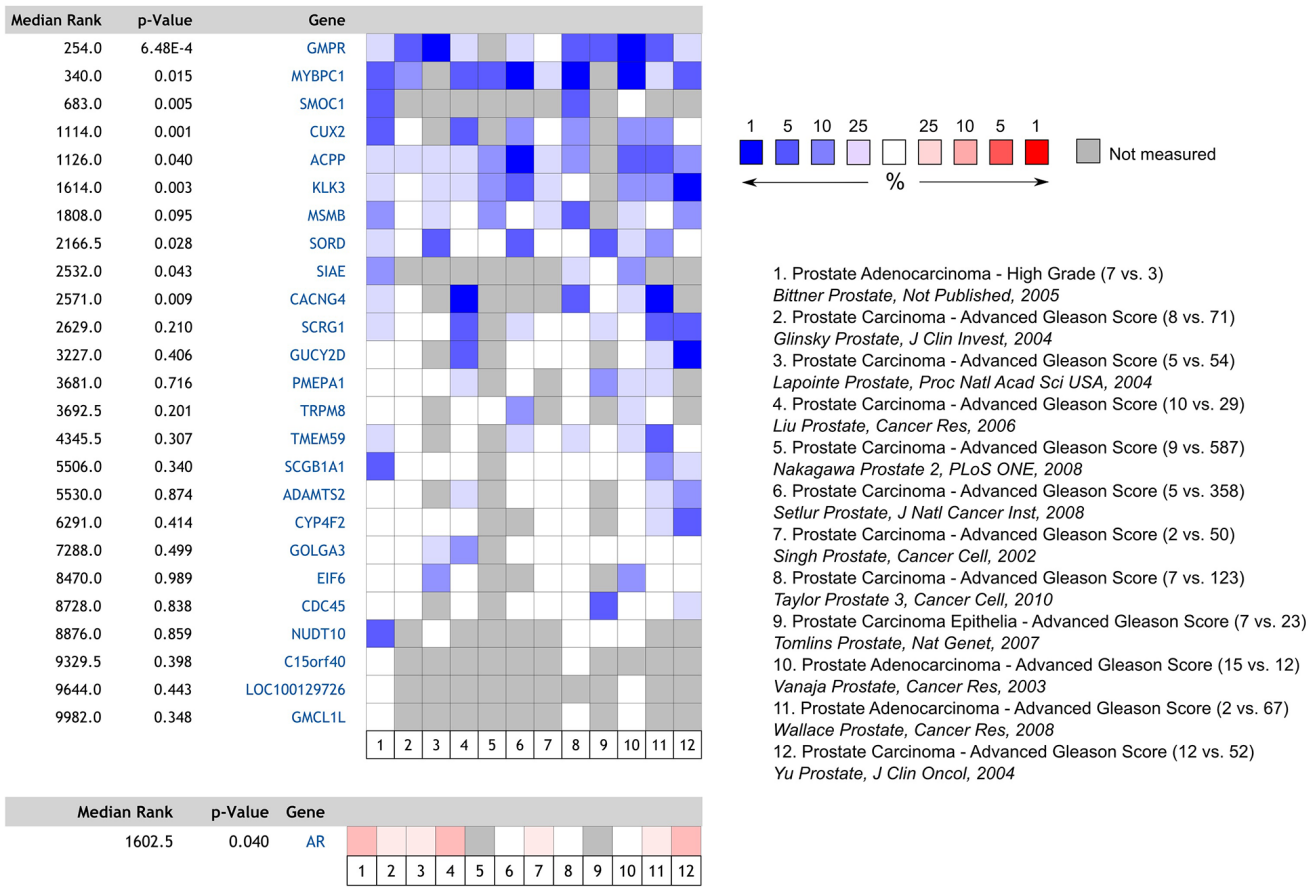
Heatmap and meta-analysis of relative androgen-regulated gene expression in high vs. low-grade prostate tumours derived from the 12 publicly accessible databases listed The number of cases in each cohort is included in parentheses (n high-grade vs. n control). Despite increased expression of AR, expression of commonly AR-regulated transcripts is decreased in high-grade disease.

To explore whether the expression of the androgen regulated transcriptome changes with increasing tumour grade, we performed a meta-analysis of the expression of the top 25 ranking androgen stimulated transcripts previously identified in benign human prostate tissue across 12 publicly accessible prostate cancer databases stratified by tumour grade (Figure 1). The listed genes encode for proteins with diverse biological functions, and includes KLK3/PSA which is a commonly used serum tumour marker which is clinically used for prostate cancer diagnosis and staging. Interestingly we observed a systematic down-regulation of normal androgen stimulated transcripts with increasing tumour grade, with nine of the 25 genes reaching the threshold for significance, despite a significant up-regulation of the androgen receptor in the same samples.

**Figure 2:**
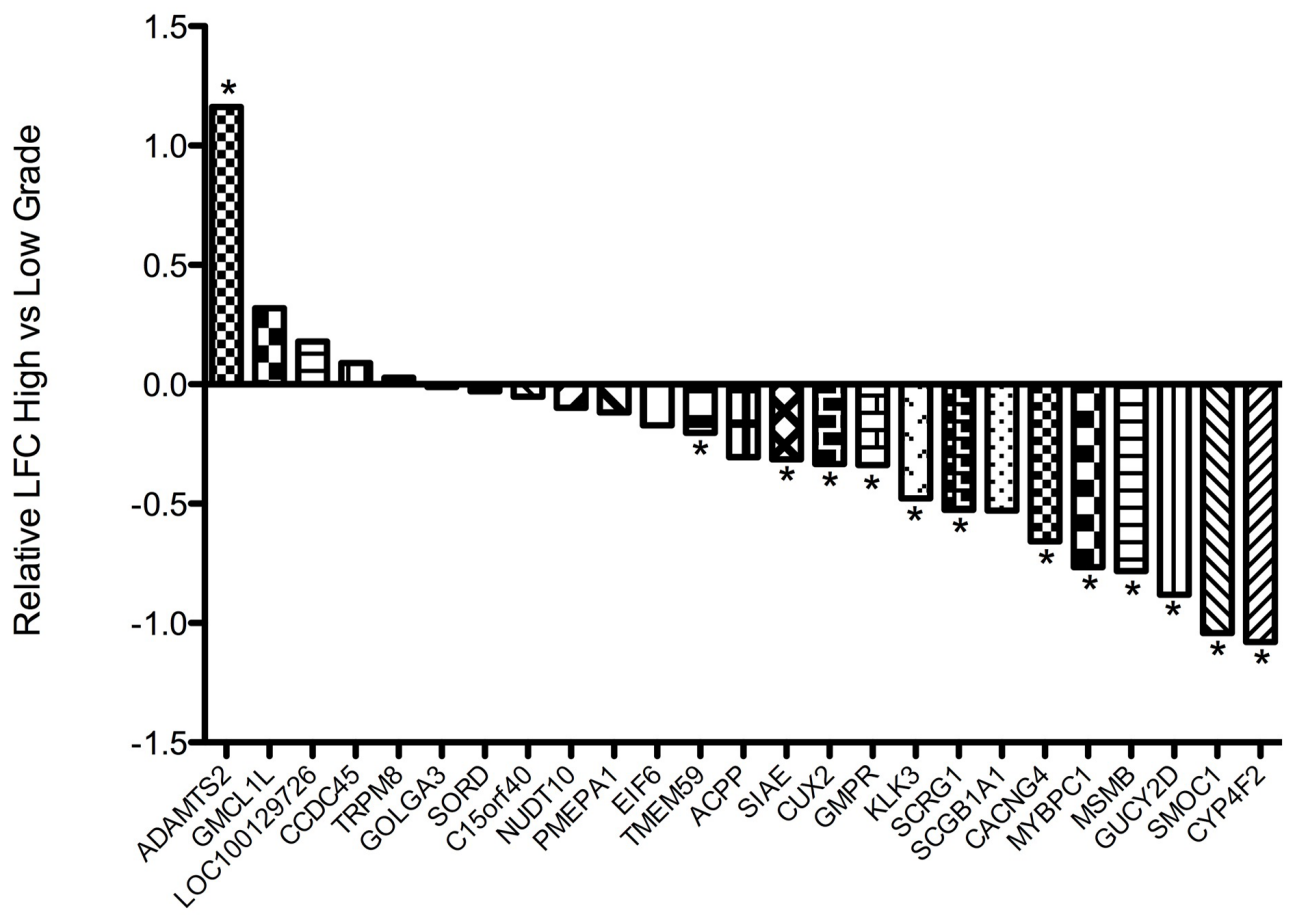
Waterfall plot depicting the relative expression of indicated transcripts in the TCGA prostate cancer dataset in high-grade (Gleason sum > 8; n = 79) compared to low-grade (Gleason sum < 6; n = 55) tumours Expression of seven genes including KLK3 was seen to be significantly down-regulated in high-grade disease. LFC = Log fold change. * False discovery rate < 0.1.

To further validate these findings, the relative expression of the same transcripts was examined in the TCGA prostate cancer expression dataset (Figure 2). When gene expression in high grade cancers was compared to that of low grade tumours, the expression of 20 of 25 genes tested were lower in higher grade tumours, and reached statistical significance for twelve, including KLK3 and MYBPC1. To determine if this decrease in the ‘normal’ AR transcriptome is associated with a concurrent change in the expression of genes specifically regulated by AR in states of altered promoter site binding, we performed a similar analyses using the AR-driven ‘M-phase cell cycle’ signature identified by Wang and colleagues as a defining feature of the transcriptome switch in androgen-independent prostate cancer [18]. Consistent with a proposed switch in the AR driven transcriptional program with local tumour progression, we observed a consistent increase in the expression of a number of these genes in higher-grade tumours, particularly CDK1, UBE2C, CDC20 and CCNA2 (Supplementary Figure S1).

**Figure 3:**
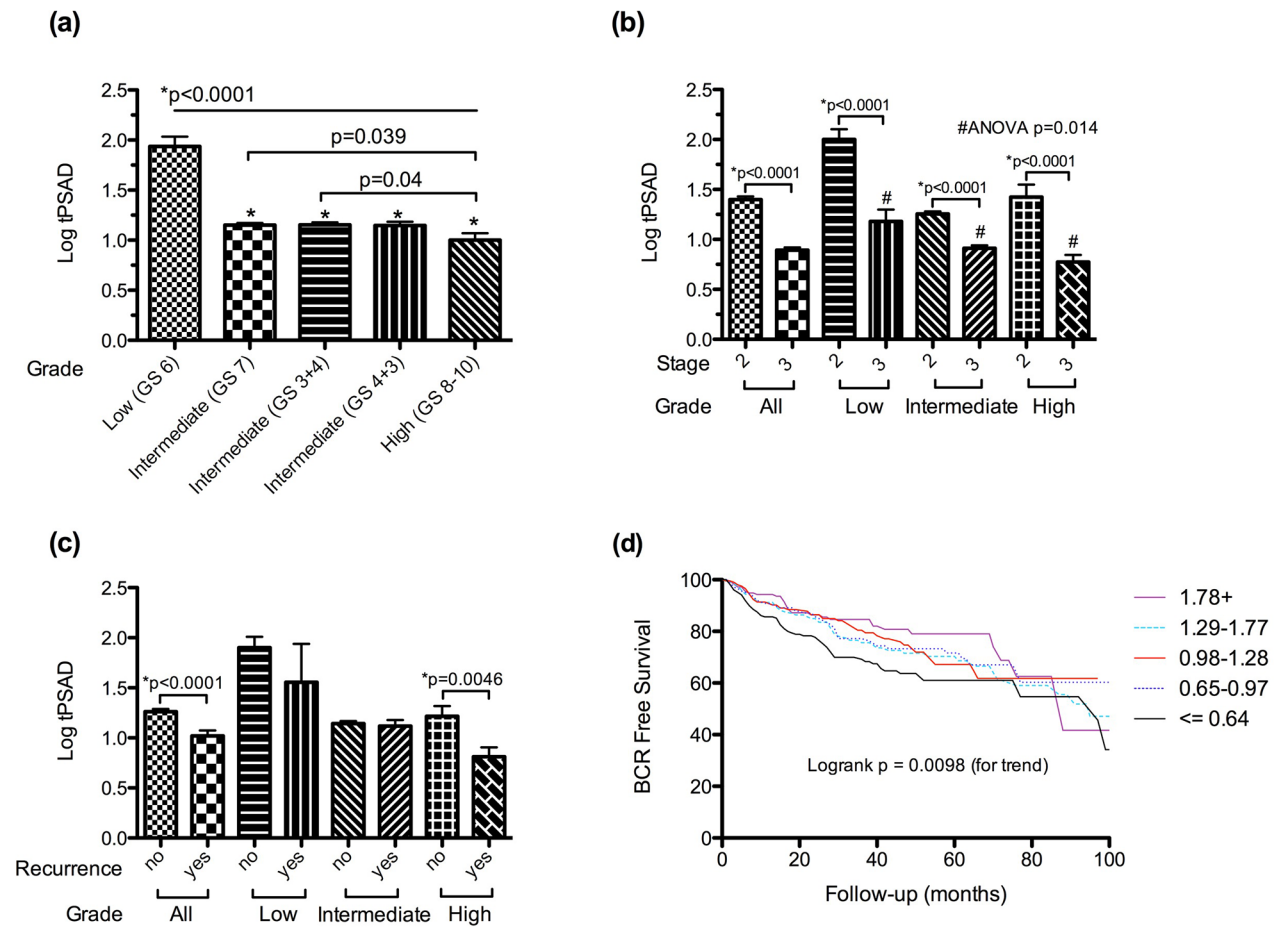
Changes in log transformed PSA per unit total tumour volume (tPSAD) with increasing **a.** tumour grade, **b.** pathological stage and **c.** the development of biochemical recurrence, stratified by tumour grade. **d.** Kaplan-Meier curve of biochemical recurrence (BCR) free survival stratified by PSA per unit tumour volume (log transformed) quintiles. Data is presented as means + SEM.

KLK3 encodes the prostate specific antigen, a serum based tumour marker that this widely used in the diagnosis and risk stratification of prostate cancer, as well as monitoring response to treatment, where higher serum levels of PSA are commonly associated with aggressive disease and poorer outcome. To determine if the observed changes in the androgen-regulated transcriptome were mirrored at the protein level, we measured serum PSA as a function of tumour volume in a large cohort of men with clinically localised prostate cancer who underwent prostatectomy (Table 1), and correlated levels with both pathological variables and clinical outcome. Consistent with gene expression, we identified a systematic decrease in serum PSA per tumour volume with increasing grade (Figure 3a) and stage (Figure 3b), with the largest incremental decreases observed at the transition points of greatest clinical interest (low grade versus intermediate grade, and organ confined versus non-organ confined). Interestingly, on univariable analysis, serum PSA per tumour volume has prognostic implications, with lower levels associated with a significantly increased risk of disease recurrence post prostatectomy (Figures 3c & 3d). However in multivariable Cox regression models including well-established clinical and pathological predictors, PSA per tumour volume was not an independent predictor, suggesting co-linearity with stage and grade (Table 2). To determine if exclusion of patients with a PSA > 20 ng/dl significantly skewed these observations, the analysis was repeated in patients irrespective of PSA with essentially identical results. In addition, similar results were obtained when index tumour volume rather than total tumour volume was used in the analysis (Supplementary Figure S2).

**Table 1:** Clinical and pathological characteristics of the study cohort

n		1108
Age (yrs)	Median	61.5
	Range	40.2-79
PSA (ng/ml)	Median	6.99
	Range	0.3-20
Pathological Stage	pT2	770 (69.6%)
	pT3	335 (30.4%)
Tumour Grade	Low (GS 6)	153 (13.8%)
	Intermediate (GS 7)	848 (76.5%)
	GS 3+4	583 (68.8%)
	GS 4+3	265 (31.3%)
	High (GS 8-10)	107 (9.7%)
Extraprostatic Extension	Absent	782 (70.6%)
	Present	326 (29.4%)
Seminal Vesicle Invasion	Absent	1042 (94.1%)
	Present	66 (6%)
Recurrence	No	812 (76.8%)
(n=1057)	Yes	245 (23.2%)
Total Tumour Volume (cc)	Median	2.6
	IQR	1.3-4.8
Prostate Volume (cc)	Median	32.2
	IQR	24.2-43.5

**Table 2:** Multivariable, Cox regression analysis of predictors of biochemical recurrence post prostatectomy

	HR	95% CIs	p
Extraprostatic extension	1.68	1.25-2.26	0.001
Seminal vesicle invasion	1.90	1.30-2.78	0.001
Positive margins	2.12	1.60-2.79	<0.001
Tumour Grade			0
Low (GS 6)	1 (Ref)		
Intermediate (GS 7)	1.97	1.06-3.67	0.032
High (GS 8-10)	5.28	2.70-10.33	<0.001
Ln(tPSAD)	1.08	0.98-1.19	0.13

**Figure 4:**
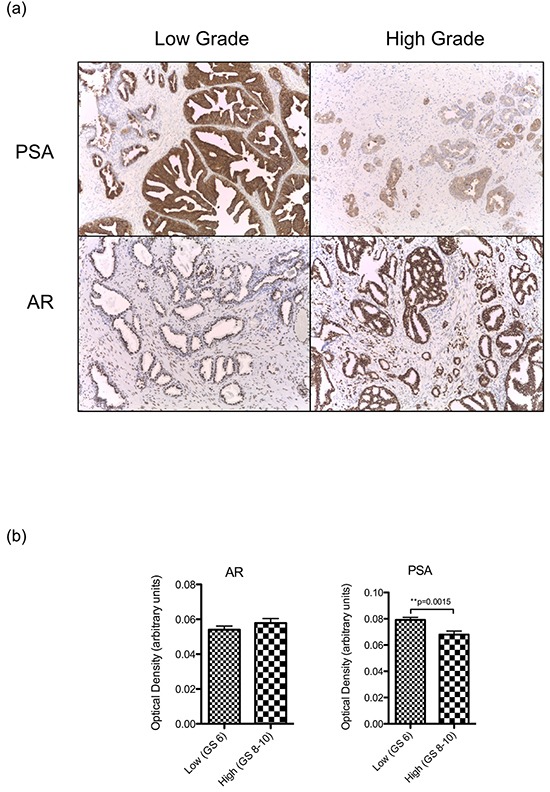
**a.** Representative images (40x) of PSA and AR expression by immunohistochemical staining in low and grade tumours. **b.** Mean + SEM expression of AR and PSA in a sample of low grade (Gleason 6, n=32) and high grade (Gleason 8-10, n=34) tumours from our clinical cohort as determined by optical densiometry of immunohistochemically stained slides. Expression between groups was compared using a 2-sided Student's t-test, with significance assumed at p<0.05.

To verify that reduced levels of KLK3 transcript are associated with a decrease in PSA production in more poorly differentiated tumours, we measured PSA expression directly in tumour sections by semi-quantitative immunohistochemistry and confirmed that expression is significantly lower in higher-grade tumours (Figure 4). There was no significant difference in AR protein expression in the same cohort.

## DISCUSSION

We have observed across multiple datasets that in comparison to low-grade disease, in high-grade disease the physiologically benign AR ‘transcriptome’ is systematically suppressed and at least for the most highly expressed AR-regulated gene, the clinically utilised tumour marker KLK3/PSA, this effect is also observed at the protein level and has prognostic relevance. As discussed above, KLK3 expression is primarily regulated by the androgen receptor, and it is a commonly used readout of the level of receptor activation both clinically and experimentally, and the most facile explanation is therefore that androgen receptor signalling is downregulated in more aggressive tumours. Receptor activation is dependent upon receptor and ligand availability, and although we do not observe a significant decrease in androgen receptor levels in the same tumour specimens, an increasing number of reports link low levels of circulating androgens to aggressive pathological features and worse outcome, although the findings are not universal [19]. However the absolute change in androgen levels are low, and certainly well above that required for full AR activation in experimental systems [20]. In addition, at least in castration-resistant cancers, tumour levels of androgen are often maintained close to normal even in the presence of castrate serum levels [21]. Another possibility is that instead of being downregulated, AR signalling is instead shifted from one transcriptional program to another. Certainly during prostate organogenesis androgens initially stimulate a program of cell proliferation, survival and mesenchymal invasion, and then switch late in the second trimester to promote epithelial cell terminal differentiation and a secretory phenotype [22]. As many of the biological programs of embryogenesis resemble those of carcinogenesis, it is possible that the observed decrease in KLK3 expression represents a transition to a more ‘developmental’ type AR transcriptional program, perhaps due changes in promoter methylation status within specific gene cassettes or the loss or gain of a critical pioneering co-transcription factor [18].

These results however do provide some insights into the clinical use of PSA as a tumour marker. PSA, being produced in significant amounts only by prostate epithelial cells, was shown in the seminal study that prompted its FDA approval to be present at higher levels in men with prostate cancer compared to ‘normal’ controls, with serum levels correlating with disease stage [23]. With the recognition that an elevated PSA was more indicative of the presence of prostate cancer than either digital rectal exam or transrectal ultrasound, its use as a community-based screening test has become entrenched. However, PSA screening has remained controversial ever since its inclusion in clinical practice and in 2012 the U.S. Preventive Services Task Force gave its final recommendation against PSA screening for men, citing “overdiagnosis” as causing more harm than the clinical benefits provided [24]. As we have demonstrated, PSA is a continuum of risk and obtaining a reading in the ‘normal’ range does not preclude the presence of a high-grade tumour due to lower PSA secretion per unit tumour volume. For instance, a 1cc organ-confined low grade tumour would increase serum PSA by a mean of 4.01 ng/ml and lead to a biopsy, whereas by comparison, a similarly sized invasive high grade tumour leads to a mean increase of only 1.71 ng/ml, which may not meet a pre-specified biopsy threshold. At the current time, tumour volume can only be accurately determined once the prostate gland has been removed. While Magnetic Resonance Imaging (MRI), the most commonly used imaging modality for local staging is reported to have high sensitivity for index tumour detection, particularly in higher-grade tumours, it routinely underestimates pathologically defined tumour volume [25–27]. However, the development of improved formulas that greatly increase the accuracy of MRI volume estimates as well as the potential of dual modality imaging with PSMA-PET raises the possibility that accurate pre-operative tumour volume estimates may be available to clinicians in the near future [19, 28]. With this in mind we suggest that there is the potential to, in the future, add tumour volume to the current repertoire of diagnostic tests alongside PSA screening and DREs to increase the reliability of the former. Our data suggest that it may even be possible to determine a threshold of tPSAD which when combined with existing prognostics, such as Gleason sum and pathological staging, will more accurately predict biochemical recurrence and allow increased care in monitoring affected patients.

In conclusion we have determined using a clinically relevant gene set that aggressive tumours have a deregulated AR transcriptome including expression of PSA and therefore aggressive, low-volume tumours may currently be overlooked by PSA screening.

## MATERIALS AND METHODS

### Meta-analysis of androgen regulated gene expression in high versus low grade prostate cancer

Genes up-regulated in benign human prostatic tissue in response to androgen stimulation were identified from a previously published study [13]. Given the low dynamic range of expression and lack of statistical significance for many of the changes identified, genes were ranked by logfold change and the top 25 genes chosen arbitrarily for analysis. A meta-analysis of individual genes across publicly accessible prostate cancer expression databases was performed using the meta-analysis tool within Oncomine (https://www.oncomine.com), the steps of which have been previously described in detail [14]. Briefly, microarray data from individual studies are normalised, and differential expression between pre-specified classes is determined by Students t-test. Genes are ranked within each dataset by the p-value, and enrichment (positive or negative) across studies determined by comparing observed rankings against a random distribution. Studies selected had a patient cohort >40 and allowed differential expression analysis by tumour grading within Oncomine. For the purposes of this analysis, high-grade tumour was classified as the group with the highest Gleason score within each individual study, with all remaining tumours acting as the comparator.

### Analysis of TCGA expression data

RNA-Seq data was obtained from TCGA and raw gene-level counts were used for the analysis. Samples were again categorised into two categories by the patient's tumour grade based upon the centralised pathology review: (i) low grade (Gleason sum ≤ 6, n=55); and (ii) high grade (Gleason sum ≥ 8, n=79). To minimise spurious statistical tests involving low expression counts, genes containing counts-per-million values (CPM) <1 for more than a third of samples were filtered out. Differential expression analysis was conducted using the edgeR Bioconductor package. Normalisation factors were calculated using Trimmed Mean of M-values (TMM) normalisation to account for differences in library size. To normalise gene-level variance, the biological coefficient of variation (BCV) was calculated using Cox-Reid dispersion for negative binomial general linear models. Samples were fitted to a negative-binomial log-linear model and tested for differential expression using a likelihood-ratio test. The p-values of differential expression tests were corrected for multiple-hypothesis testing using Benjamini-Hochberg false discovery rate (FDR) correction. The threshold for significance was set to FDR<0.1.

### PSA patient cohort selection

Consecutive patients with a diagnosis of primary prostate cancer, localised to the prostate, who underwent surgical removal of their prostates at the Epworth Hospitals in Melbourne from 2003 to December 2011 and had detailed PSA follow-up were identified from a prospectively recorded and maintained dedicated prostate cancer database (n=1228). Nine patients with missing pre-operative serum PSA values were excluded from the cohort. To minimise the impact of occult metastatic disease and large prostate gland volume on the analysis, patients with a pre-operative PSA > 20 ng/ml (n=55) or with a gland weight above the 95th percentile (84 g, n=56), were excluded from the analysis. Samples were divided into three groups by Gleason sum to include low grade (GS ≤ 6), intermediate grade (GS = 7), and high grade (GS ≥ 8). Collection and use of tissue and data had individual patient consent and institutional review board approval.

### Calculation of tumour volume

As part of routine pathological assessment, tumour volume was calculated in all patients as previously described [15]. Briefly, transverse whole-mount sections of formalin fixed and paraffin-embedded surgical specimens were taken at 3.5 mm intervals perpendicular to the urethra from prostate base to apex, and slides stained with haematoxylin and eosin. After tumour borders were outlined manually with a pen, slides were digitalised and tumour volumes calculated using image analysis software. To account for formalin induced shrinkage a correction factor of 1.25 was applied. Prostate dimensions were measured and gland volume calculated using the ellipsoid formula.

### Calculation of tumour attributable serum PSA and PSA per unit tumour volume

The volume of the prostate gland occupied by benign tissue was calculated by subtracting total tumour volume from total prostate volume measured after the prostate had been surgically removed. In seven cases where the tumour comprised the whole of the prostate, the benign volume was set to zero. The contribution of benign prostate tissue to serum PSA was determined by the following calculation: PSA from benign tissue = (epithelial fraction) x (PSA/cm^3^ epithelial tissue) x (volume of benign prostate tissue). The epithelial fraction was set at 0.1 and the PSA production of benign epithelial tissue set at 0.30 ng/cc [16, 17]. The amount of serum PSA contributed by cancer was determined by subtracting ‘benign PSA’ from the total serum PSA. The amount of PSA produced per cc of tumour (tumour PSA density, tPSAD) was derived by dividing ‘cancer PSA’ by total tumour volume. To meet normality assumptions, PSA per tumour volume measurements were log transformed prior to analysis. Data are presented as medians and ranges or means + SEM as appropriate, and differences between groups analysed by t-test or ANOVA as indicated. All statistical analyses were performed using Graphpad Prism V5.

### Immunohistochemistry

Immunohistochemistry (IHC) was performed using an automated immunostainer (Ventana Medical Systems, Tucson, Arizona). For full-length androgen receptor staining, 5 micron FFPE sections were treated with pre-diluted CC1 antigen retrieval buffer for 64 minutes and incubated with anti-AR antibody (Cell Marque SP107 clone, Ventana) at a dilution of 1:20 for 60 mins at 40°C. For PSA staining, antigen retrieval was in the same buffer but for 36 minutes, followed by incubation with anti-PSA antibody (NCL-PSA-431, Leica) at 1:200 dilution for 8 mins at 36°C. Antibody binding was detected with the ultraView Universal DAB Detection Kit (Ventana) followed by manual counterstaining with haematoxylin. For digital image analysis, images of stained slides were taken at 20x magnification, and uploaded onto the Fiji version of ImageJ. Images were converted into an 8-bit format, and colour deconvoluted with H-DAB as the stain. Regions of the image of equal size were selected at random in triplicate, and the mean intensity was measured. The optical density (OD) was calculated using the following formula: OD= LOG (max intensity/mean intensity), where the max intensity for an 8-bit image is 255.

## SUPPLEMENTARY FIGURES


