# Supplementary material for: Reduction in expression of the benign AR transcriptome is a hallmark of localised prostate cancer progression
Source: Oncotarget. 2016 Apr 22;7(21):31384–92. doi: 10.18632/oncotarget.8915 (PMC5058764; doi:10.18632/oncotarget.8915)
Supplement: Supplementary file 1 [file oncotarget-07-31384-s001.pdf]

SUPPLEMENTARY FIGURES

(a)

Median Rank	p-Value	Gene
787.0	0.014	CDK1
1487.0	0.059	UBE2C
1870.0	0.078	CDC20
1901.5	0.090	CCNA2
7285.0	0.487	ID1
8095.0	0.314	ANAPC10
9355.5	0.710	PRDM4

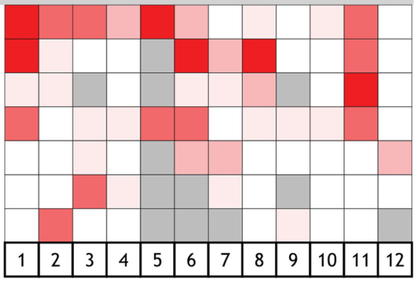

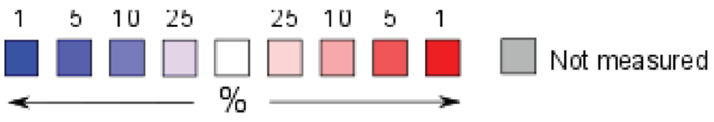

(b)

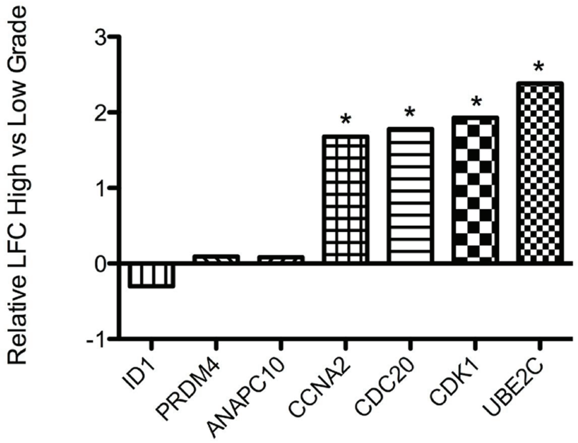

Supplementary Figure S1: a. Heatmap and meta-analysis of the specific androgen independent, AR regulated M-phase cell cycle genes, in in high vs. low-grade prostate tumours derived from the 12 publicly accessible databases listed. The listed studies are the same as in Figure 1. **b.** Waterfall plot depicting the relative expression of indicated transcripts in the TCGA prostate cancer dataset in high-grade (Gleason sum > 8; n = 79) compared to low-grade (Gleason sum < 6; n = 55) tumours. LFC = Log fold change. * False discovery rate < 0.1.

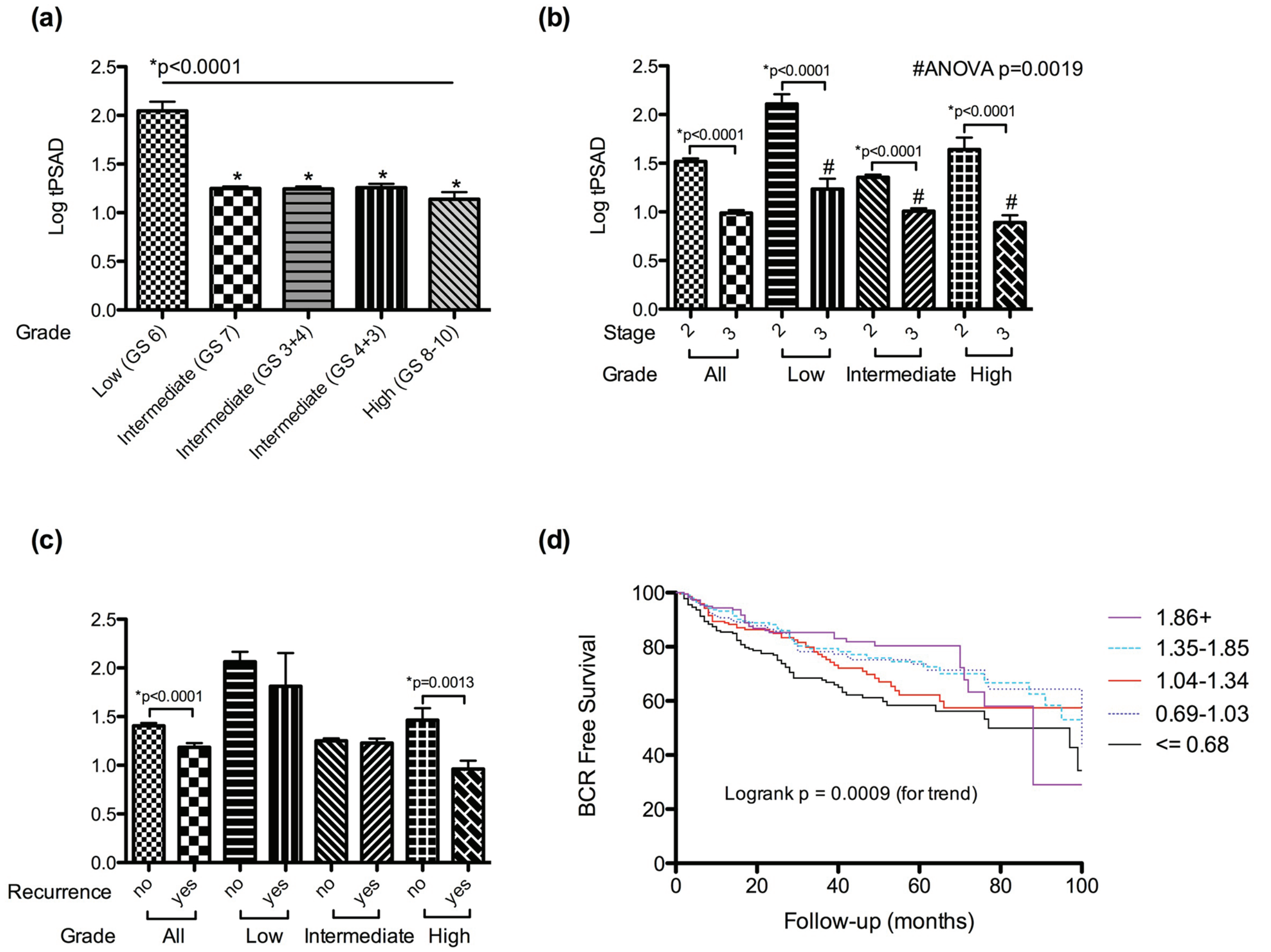

Supplementary Figure S2: Changes in log transformed PSA per unit index tumour volume (tPSAD) with increasing **a.** tumour grade, **b.** pathological stage and **c.** the development of biochemical recurrence, stratified by tumour grade. **d.** Kaplan-Meier curve of biochemical recurrence (BCR) free survival stratified by PSA per unit tumour volume (log transformed) quintiles. Data is presented as means + SEM.
